# *Vibrio vulnificus* infection attributed to bee sting: a case report

**DOI:** 10.1080/22221751.2021.1977589

**Published:** 2021-09-17

**Authors:** Jie-Heng Liang, Wen-Huan Liang, Yun-Qi Deng, Zhi-Gang Fu, Jun-Li Deng, Yong-Hua Chen

**Affiliations:** The Seventh People’s Hospital of Nanhai, Foshan, Guangdong, People's Republic of China

**Keywords:** *Vibrio vulnificus*, bee sting, infection, insect-borne transmission, Sepsis

## Abstract

*Vibrio vulnificus* is a pathogenic marine bacteria associated with high mortality. Changes in climate and the global seafood trade have increased the prevalence of marine and freshwater systems affected by *V. vulnificus*. As a result, the incidence of land animals, plants, and insects contacting *V. vulnificus* and acting as disease vectors is on the rise. We report the case of a 53-year-old male who was infected with *V. vulnificus* as the result of a bee sting. The patient had no history of contact with the sea or fresh water or aquatic organisms or products. Due to bacterial pathogenicity and the patient’s underlying diseases, his condition deteriorated rapidly and eventually resulted in death. Here, we review the pathogenic mechanisms and treatment of *V. vulnificus*. We determined that *V. vulnificus* has spread from seawater to freshwater and that individuals may become infected from insects, even in the absence of direct contact with infected water. This case report will inform clinicians about the possible sources of *V. vulnificus* infection and indicates the possibility that more insects may transmit *V. vulnificus* in the future.

## Background

*Vibrio vulnificus* is a marine pathogen associated with high mortality. The primary clinical manifestations of *V. vulnificus* infection include acute fever, chills, haemorrhagic bullous lesions, and septic shock. Severe cases of *V. vulnificus* infection can quickly lead to death. Even in the United States, the mortality of *V. vulnificus* infection is as high as 50%, according to the FDA [1]. Patient prognosis largely depends on early diagnosis and treatment. Disease can occur as little as 4 h after ingestion or exposure to *V. vulnificus*, and the mortality rate of patients without proper treatment within three days can reach 100% [2]. *V. vulnificus* is a halophilic and thermophilic bacteria; when the water temperatures exceed 18°C, *V. vulnificus* propagation increases, with an optimum growth temperature of 26°C [3]. As a result of increasing sea surface temperatures driven by climate change, the endemic area of *V. vulnificus* is expanding annually [4]. Additionally, the increase of seafood aquaculture and global seafood trade has resulted in increasing reports of *V. vulnificus* in freshwater environments [5–7]. As *V. vulnificus* colonizes freshwater environments, the possibility of terrestrial animals, plants, and insects being exposed to *V. vulnificus* and potentially becoming disease-transmitting vectors concomitantly increases. The atypical clinical signs of *V. vulnificus* infection along with increasingly unclear exposure risk factors have caused difficulties in its early diagnosis and treatment.

## Case report

**Figure 1.**
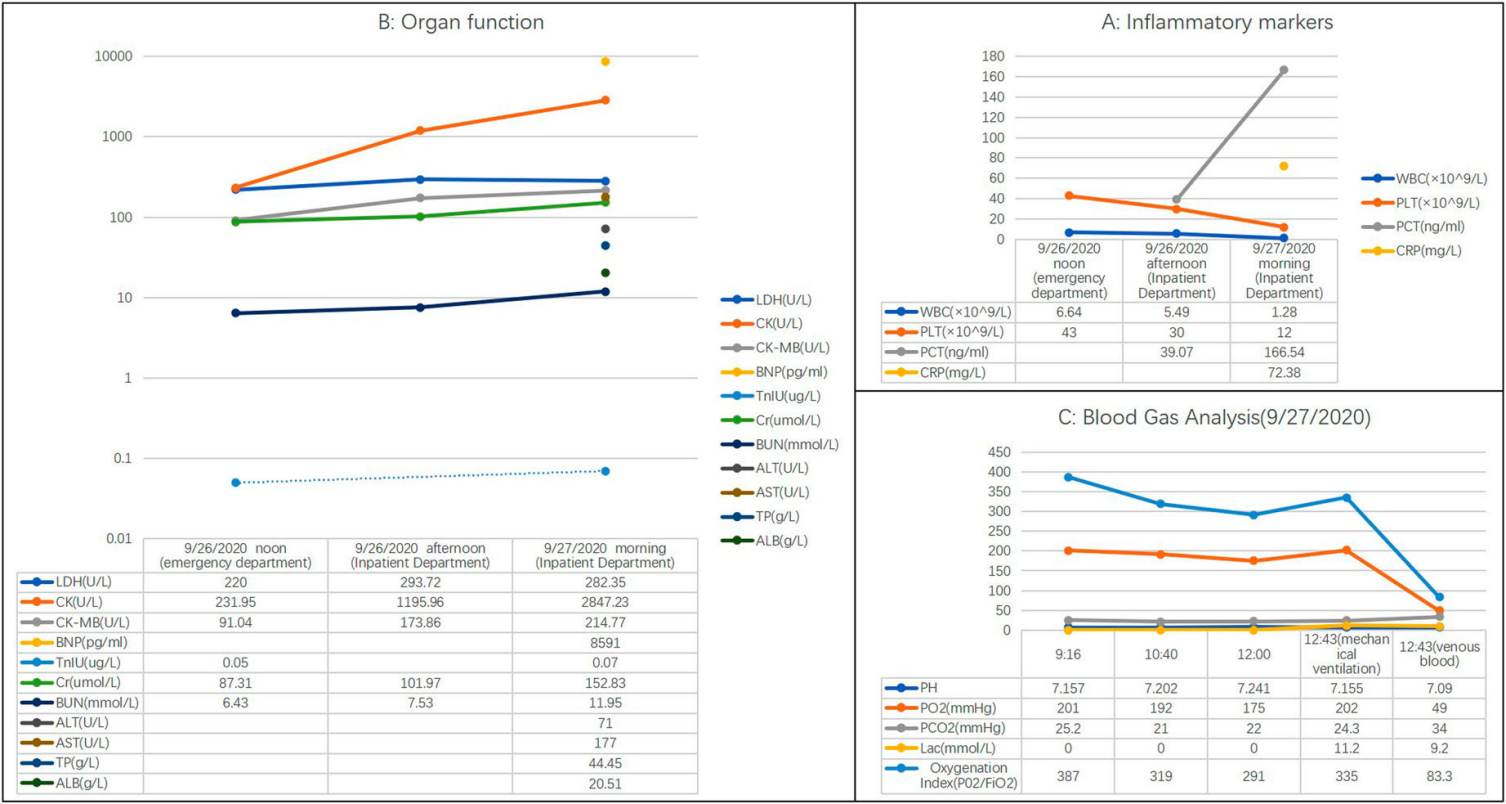
Blood test results of this patient. WBC and PLT levels continued to decline, PCT continued to rise (A). Function of vital organs including, the heart, kidney, and liver, was abnormal and continued to deteriorate (B). Acid base imbalance, lactic acid accumulation, and oxygenation index decreased until mechanical ventilation was initiated (C).

**Figure 2.**
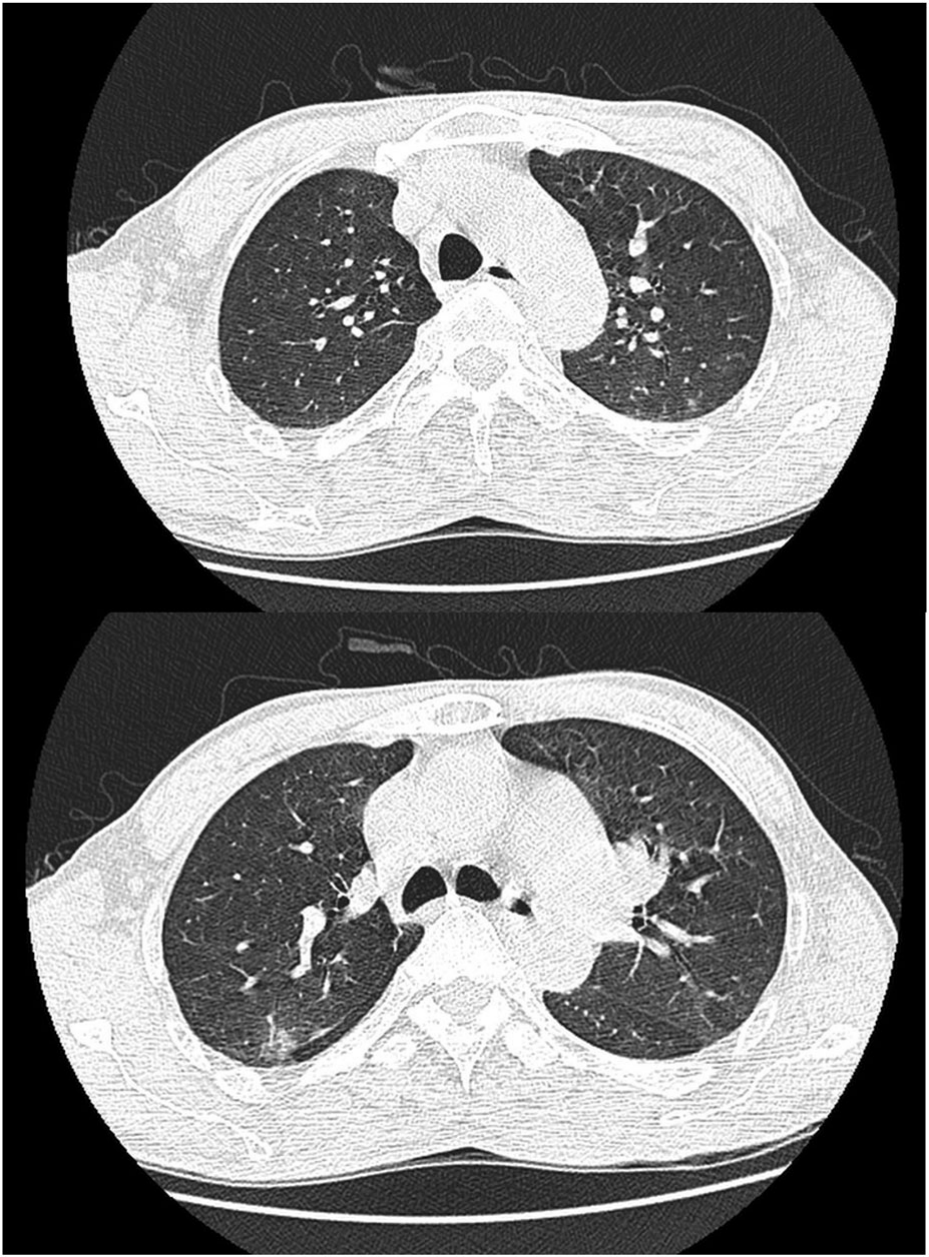
inflammation in the posterior segment.

**Figure 3.**
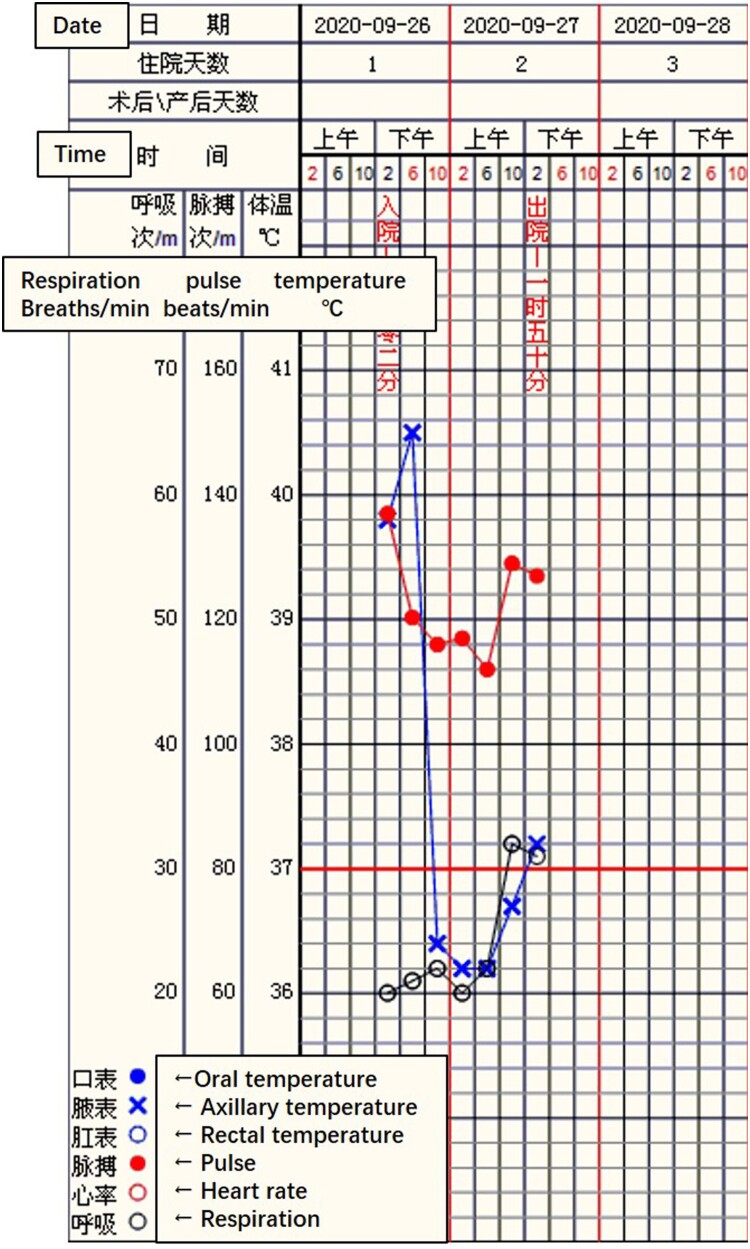
Vital sign of this patient: The heart rate was above 110 bpm, and the highest body temperature was 40.5°C before taking NSAID.

A 53-year-old male was admitted to Seventh People’s Hospital of Nanhai on 26 September 2020, presenting with fever after being stung by a bee the previous day. He was a freight driver and was stung by a bee while trying to pick a *Ganoderma lucidum* on a trip through Huadu. He was questioned to assess his recent exposure to possible infective agents and denied transport or exposure to aquatic products, oceans, lakes, etc. His medical history included hepatitis B cirrhosis, hyperthyroidism, chronic gastritis, and renal calculi. His highest recorded body temperature was 39.8°C, and it was accompanied by chills, vomiting, and diarrhoea. Physical examination revealed swelling of the left lower limbs and a wound from a bee sting without blisters or ulcers. Preliminary blood tests (26 September 2020 noon and afternoon) revealed significantly elevated procalcitonin (PCT), creatine kinase (CK), and CK-MB; significantly decreased platelet count (PLT), and gradually increasing blood urea nitrogen (BUN) and serum creatinine (Cr) (Figure 1(A,B)). These results indicated infection, muscle damage, and a gradual decrease in renal function. Troponin I (TnIU) was slightly increased, but there were no typical symptoms indicative of angina pectoris or ECG manifestations. Thus, evidence for the diagnosis of myocardial infarction was insufficient. Additionally, a chest computed tomography scan revealed inflammation in the posterior segment of both upper lobes (Figure 2). Nevertheless, the patient’s vital signs were stable, except for an elevated heart rate due to a high fever (Figure 3). Based on the above, the physician diagnosed hypersensitivity pneumonitis, Hepatitis B cirrhosis, secondary thrombocytopenia, and bee sting. The patient was administered antiallergic treatment to treat the bee sting. The patient had a history of insect stings and a significant rise in PCT but no corresponding rise in white blood cells. The physician failed to rule out sepsis caused by Gram-negative or anaerobic bacteria and prescribed cefotaxime sodium and sulbactam 3 g q8h as antibiotic treatment.

**Figure 4.**
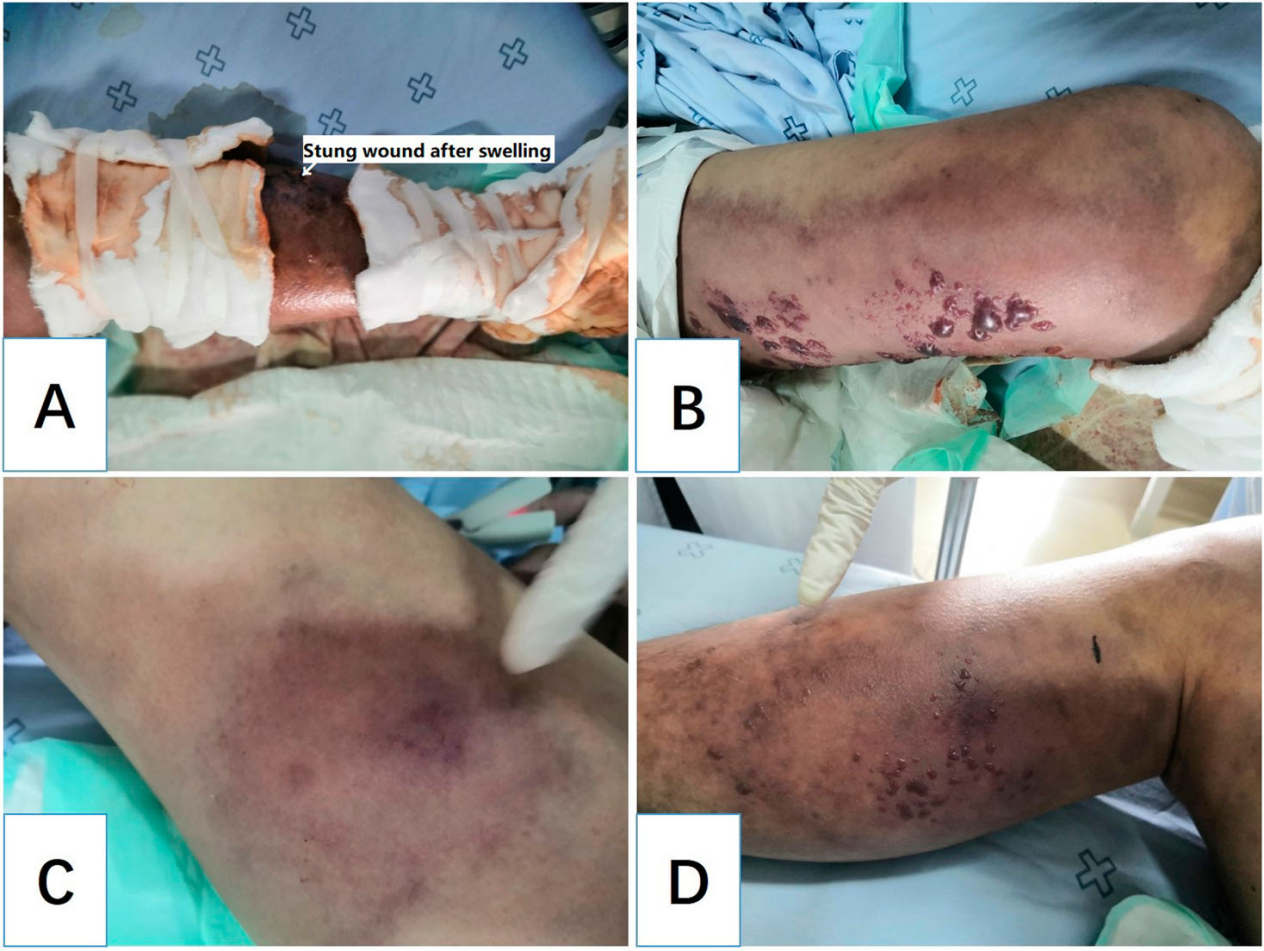
Changes in the physical signs of the patient. Cyanosis, multiple blisters, and ecchymosis around the sting wound (A). Cyanosis, multiple blisters, and ecchymosis spread to the left thigh (B). Similar cyanosis, multiple blisters, and ecchymosis appeared on the right lower limb (C and D).

**Figure 5.**
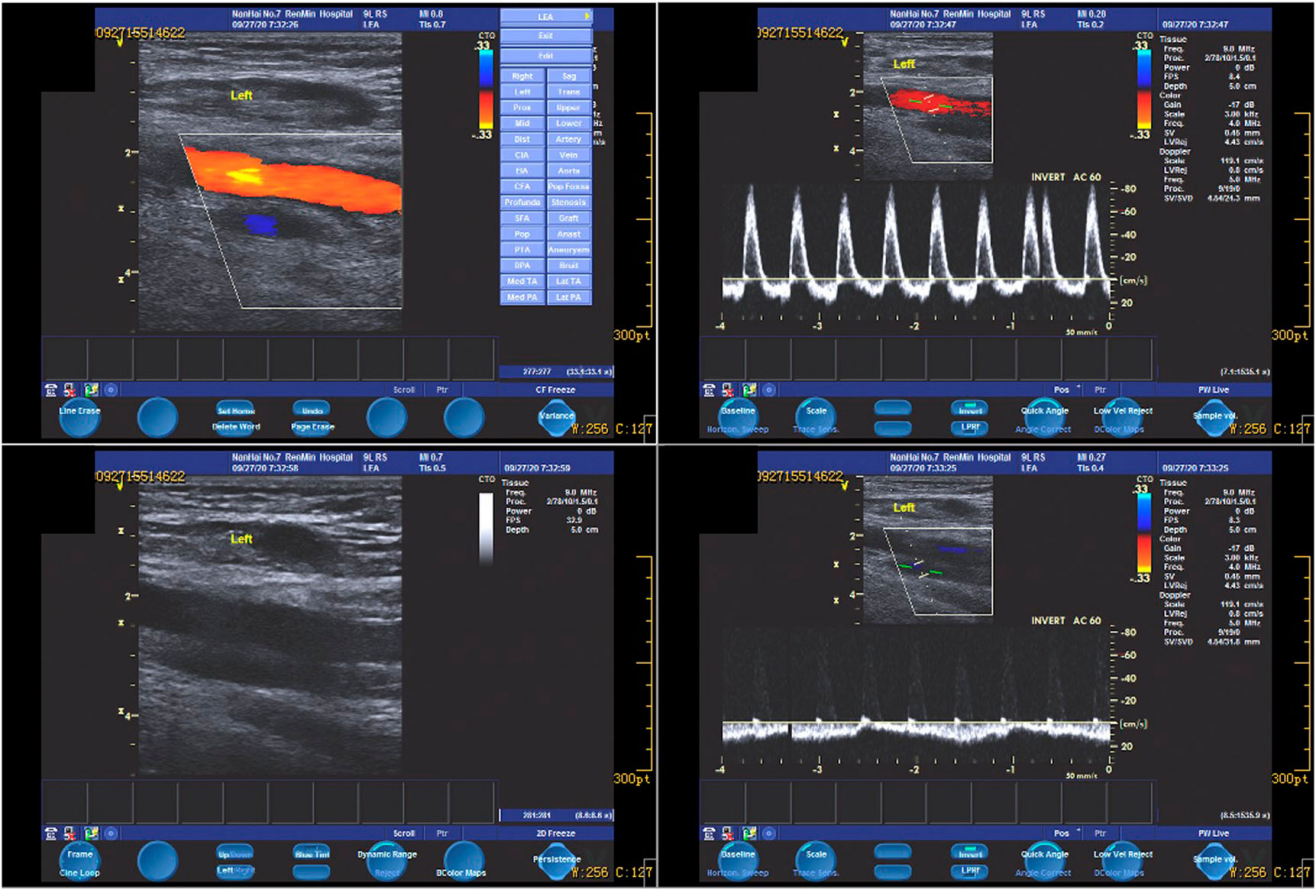
Colour Doppler ultrasound image: Deep vein blockage in left lower limb.

**Figure 6.**
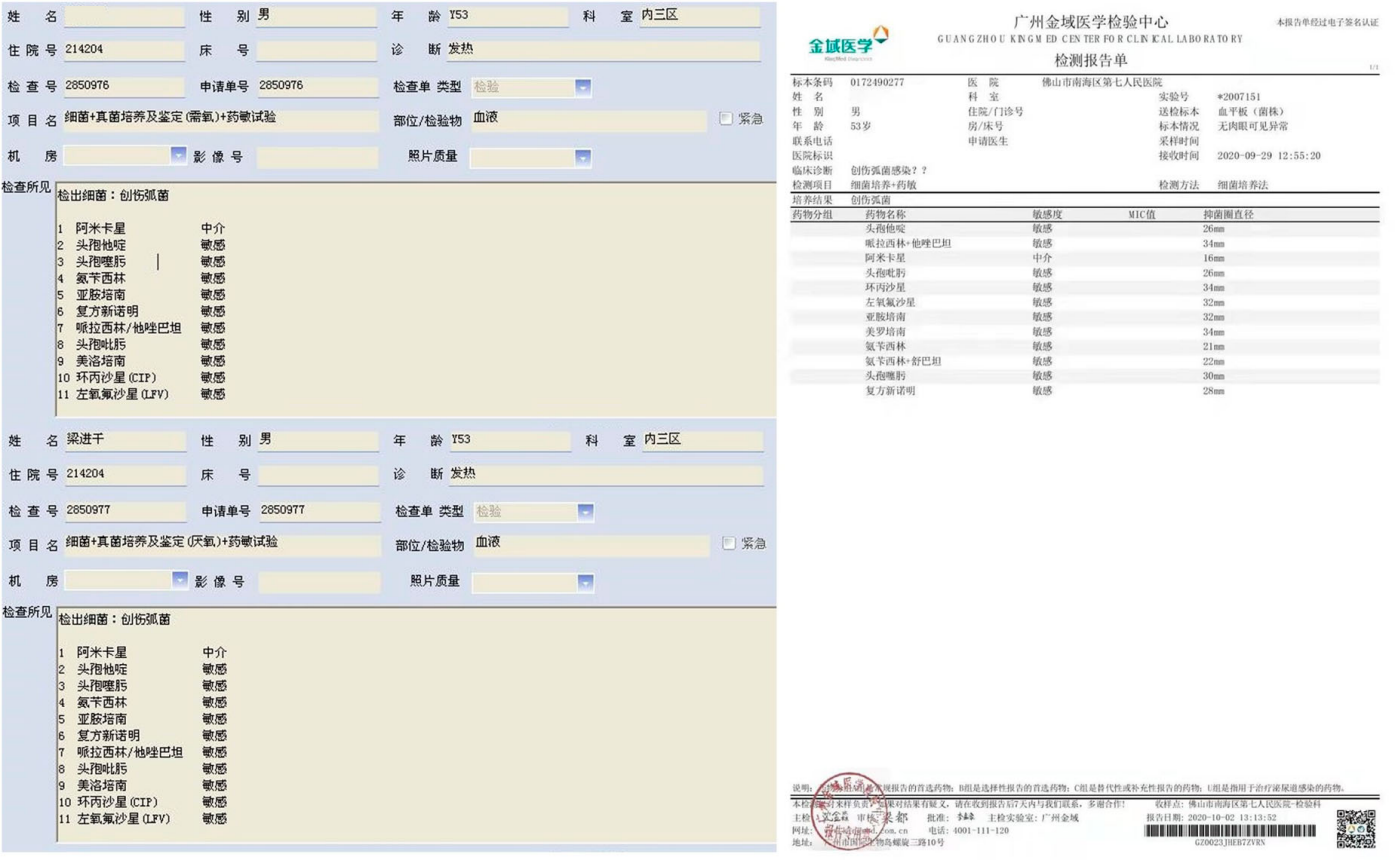
Blood culture results. Left: The culture results of two blood samples from our hospital, including aerobic and anaerobic. Right: Bacterial identification results from a third-party authority. “创伤弧菌” means “*Vibrio vulnificus*.”

Unfortunately, the treatment was ineffective, and the patient’s condition deteriorated rapidly within the next 12 h. At 5:00 in the morning following admission, the patient complained of severe pain in the left lower limb. Physical examination revealed swelling, and the left lower limb was obviously cyanotic and had multiple blisters and regions of ecchymosis scattered below the knee joint (Figure 4(A)). Moreover, ecchymosis also appeared in the right lower limb (Figure 4(C)), and the pulsation of the dorsalis pedis arteries of both lower limbs was weakened. However, the patient’s vital signs remained stable: his temperature was 36.2°C; blood pressure, 108/82 mmHg; respiration, 20 breaths/min; heart rate, 112 bpm; and SpO_2_, 98%. Three hours later, the patient developed shortness of breath and lost consciousness. At this time, the following vital signs were observed: blood pressure, 80/50 mmHg; respiration, 30 breaths/min; heart rate, 122 beats/min; and SpO_2_, 98%. Physical examination revealed that the patient’s skin was cold and wet. Swelling, ecchymosis, and blistering of the left lower limb continued to aggravate (Figure 4(B)), and blisters of the same nature appeared on the right lower limb (Figure 4(D)). Colour Doppler ultrasound of the lower limbs showed thrombosis from the left external iliac vein to the posterior tibial vein. Most of these thrombi resulted in venous blockage (Figure 5). No obvious abnormality was found in the artery or vein of the right lower limb. The patient’s laboratory test results revealed further decreased WBC (1.28 × 10^9^/L) and PLT (12 × 10^9^/L) and a continuous increase of PCT (166.54 ng/mL), indicating that the infection was further aggravated and unaffected by the antibiotic treatment. BNP was as high as 8591 ng/mL, transaminase was increased, and Cr and BUN continued to rise, indicating that the patient was experiencing multiple organ dysfunction syndrome affecting the heart, liver, and kidney. (Figure 1(A,B), 27 September 2020). The results of blood gas analysis at different periods also indicated disease progression and further deterioration of the patient’s condition. The accumulation of lactate particularly indicated a very poor prognosis. (Figure 1(C)). The physician revised the initial diagnosis to septic shock, diffuse intravascular haemolysis, multiple organ dysfunction syndrome, and bee sting. During rescue treatment, mechanical ventilation was implemented to improve the ventilation of the patient, low molecular weight heparin was administered for anticoagulation, norepinephrine was administered to maintain blood pressure, and plasma and fluid resuscitation were infused. However, the patient’s condition continued to deteriorate, and he eventually died, fewer than two days following admission. A few days later, the laboratory cultured *V. vulnificus* from blood samples of the patient (Figure 6).

## Literature review

*V. vulnificus* is a halophilic, marine, Gram-negative bacteria that primarily inhabits marine environments. Because of its high pathogenicity and mortality, it has become the leading cause of death related to seafood consumption in the United States. In China, most cases of *V. vulnificus* infection are reported in the southeast coastal areas [8]. The high pathogenicity and lethality of *V. vulnificus* infection is due to the presence of cytolysin, metalloproteinase, and capsular polysaccharides. Together, these virulence factors trigger an immune response. Iron overload was a high-risk factor in this patient, who had a history of hepatitis B cirrhosis. On its own, hepatitis B cirrhosis can lead to decreased hepcidin levels, resulting in the overexpression of ferroportin (FPN), causing iron overload [9]. Iron overload can significantly increase the pathogenicity of *V. vulnificus* [10].

Early diagnosis and early start of multi-disciplinary treatment are the key to the treatment of *V. vulnificus* sepsis. Antibacterial treatment should follow the principle of early, combined, and sufficient loading dose [11]. In severe infection, tetracyclines and carbapenems are less effective, and third-generation cephalosporins combined with quinolones are the best choice [12–14]. Therefore, once an early diagnosis is established, the patient should be treated with third-generation cephalosporins and quinolones for 7–10 days [11,15].

## Discussion and conclusion

Since both the patient and his family denied any history of contact with seawater, freshwater, or marine organisms, and the patient only had an acute history of bee sting, the early diagnosis was misjudged as bee sting poisoning and allergies. However, through the results of post-mortem blood cultures and a review of the patient’s condition, infection of *V. vulnificus* was evident. Humans infected with *V. vulnificus* generally have exposure histories to water systems or aquatic products. We have repeatedly confirmed the relevant exposure history with the patient and his family, but they always gave a denial reply. These exposure histories are not shameful or interest related. They have no motive and reason to conceal. We could not identify other possibilities leading to *V. vulnificus* infection. Therefore, the focus of this discussion concerns the transmission of *V. vulnificus* through insects such as bees.

Some subspecies of *V. vulnificus* can survive in freshwater environments [16]. Lei et al. studied the presence of *V. vulnificus* in fresh water products from Guangdong, Guangxi, Beijing, Shenzhen and other places in China, and found that *V. vulnificus* infection was present in multiple species and was characterized by easy infection and seasonality [17]. In Guangdong province, Yuan et al. detected *V. vulnificus* in freshwater fish in Shenzhen, and the detection rate in some freshwater fish was up to 38.6%, which is higher than that of seafood. The positive detection rate in fresh water was 19.9% [18]. There are also clinical reports of *V. vulnificus* infection after exposure to fresh water. Zhongshan Hospital of Fudan University reported a case of *V. vulnificus* infection related to freshwater shrimp stings [6]. The above studies show that fresh water has been colonized by *V. vulnificus*, which is far more serious than expected. Through infected freshwater or aquatic product, *V. vulnificus* can infect human beings.

Insect bites can lead to severe infections. Olivares-Becerra et al. reported the first case of the association between spleen abscess and bee bite [19]. Richardson and Schmitz reported a case of chronic relapsing cervicofacial necrotizing fasciitis caused by a bee sting [20]. Shahar et al. reported a case of *Pseudomonas aeruginosa* arthritis and osteomyelitis of the foot in a boy whose foot had been stung by a bee [21]. However, these case reports did not analyze the pathogenesis. Truskinovsky et al. tried to propose several possible mechanisms, and he believed that bee sting was the key. Through the stinging mechanism of bees, any bacteria on either the bee's body or its sting, can be inoculated under the epidermis [22].

Bees collect gum, water, and inorganic salts in addition to pollen and nectar during foraging. Bees forage on a relatively large range, about 1 km in radius, but in the absence of adequate nectar sources, this range can reach 4 km [23]. Bees build a static electric charge during the flight due to high-speed vibration of their wings and friction with the air [24]. This charge can cause small particles and materials to adsorb to the surface of the bee. In the process of collection, bees will adsorb toxic or polluting substances and bacteria from different environments such as air, water, soil, and plants. These substances can then be carried back to the hive [25]. Bees have ample opportunity to contact fresh water infected by *V. vulnificus* and adsorb bacteria onto their bodies through static electricity. When attacking a human, *V. vulnificus* can then enter the body through the bee sting site. In addition, patients with liver diseases who accumulate large amounts of iron in the liver are at risk for worsened *V. vulnificus* infection. Under these conditions, *V. vulnificus* grows rapidly through iron overload and causes a series of bodily injuries and may eventually lead to death.

This unique case indicates that *V. vulnificus* has spread from seawater to freshwater and that individuals may become infected even in the absence of direct contact with infected water. Clinicians treating high-risk patients should enhance vigilance and consider atypical routes of *V. vulnificus* infection, especially in patients likely to develop severe cases. In suspected cases of *V. vulnificus*, early intervention with appropriate intravenous antibiotics is critical in order to reduce the mortality and disability rate.
